# Attitudes of patients with cutaneous melanoma toward prognostic testing using the 31‐gene expression profile test

**DOI:** 10.1002/cam4.5047

**Published:** 2022-08-01

**Authors:** Kelli Ahmed, Jennifer J. Siegel, Sonia K. Morgan‐Linnell, Kyleigh LiPira

**Affiliations:** ^1^ Castle Biosciences, Inc. Friendswood Texas USA; ^2^ Melanoma Research Foundation Washington District of Columbia USA

**Keywords:** 31‐GEP test, cancer, gene expression profiling, melanoma, oncology, prognosis, surveys and questionnaires

## Abstract

**Objective:**

Although most patients diagnosed with early‐stage cutaneous melanoma (CM) have excellent outcomes, because of the large number diagnosed each year, many will experience recurrence or death. Prognostic testing for CM using the 31‐gene expression profile (31‐GEP) test can benefit patients by helping guide risk‐appropriate treatment and surveillance plans. We sought to evaluate patients' attitudes toward prognostic testing with the 31‐GEP and assess whether patients experience decision regret about having 31‐GEP testing.

**Methods:**

A 43‐question survey was distributed by the Melanoma Research Foundation in June–August 2021 to CM patients enrolled in their database. Patients were asked questions regarding their decision to undergo 31‐GEP testing and the extent to which they experienced decision regret using a validated set of Decision Regret Scale questions.

**Results:**

We analyzed responses from patients diagnosed in 2014 or later (*n*  = 120). Of these, 28 had received 31‐GEP testing. Most respondents (*n*  = 108, 90%) desired prognostic information when diagnosed. Of those who received 31‐GEP testing, most felt the results were useful (*n*  = 22 out of 24) and had regret scores significantly less than neutral regret, regardless of their test results (Class 1: *p*  < 0.001; Class 2: *p*  = 0.036). Further, decision regret scores were not significantly different between patients who received a Class 1 31‐GEP result and those who received a Class 2 result (mean Class 1 = 1.39 and mean Class 2 = 1.90, *p*  = 0.058).

**Conclusions:**

Most newly diagnosed CM patients desired prognostic information about their tumors. Patients who received 31‐GEP testing felt it was useful and did not regret their decision to undergo 31‐GEP testing.

## Graphical abstract

Prognostic testing for CM using the 31‐gene expression profile (31‐GEP) test can benefit patients by helping guide risk‐appropriate treatment and surveillance plans. A 43‐question survey regarding their decision to undergo 31‐GEP testing and the extent to which they experienced decision regret was distributed by the Melanoma Research Foundation to CM patients. Most newly diagnosed CM patients desired prognostic information about their tumors. Patients who received 31‐GEP testing felt it was useful and did not regret their decision to undergo 31‐GEP testing.

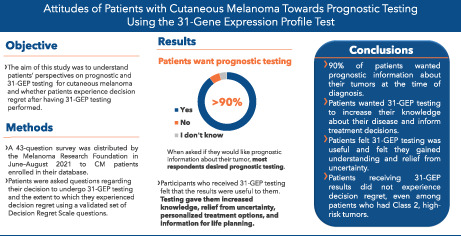

## 1 INTRODUCTION

In the United States, cutaneous melanoma (CM) is the 5th most common cancer, with over 100,000 new cases diagnosed and 7000 deaths annually.^1^ Most patients newly diagnosed with CM are classified as low risk based on American Joint Committee on Cancer (AJCC) staging guidelines (Stage I or Stage II)^2^ and have excellent prognoses; however, up to 44% of Stage II patients will experience recurrence, and many will die of their disease.^3^, ^4^

Prognostic assessment and risk stratification are used to guide treatment plan decisions in CM, be it clinicopathologic factors alone or in combination with gene expression profile testing. It is important to incorporate more accurate prognostic testing methods to identify patients at high risk of poor outcomes, particularly because planned imaging studies are effective at identifying metastatic spread early and there are now effective adjuvant therapies available for patients with CM. Similarly, patients with a low risk of poor outcomes can avoid an unnecessary sentinel lymph node biopsy surgical procedure.

It has been suggested that a poor prognosis could potentially cause patients to regret their decision to undergo prognostic testing; however, few studies have formally assessed decision regret levels after prognostic testing for CM.^5^, ^6^ However, studies of medical decisions for other cancer types (e.g., breast, prostate) and diseases have found that patients who felt their treatment options were clear and well‐explained by their physician and who felt involved in their treatment‐ and care‐related decisions experience less regret.^6^, ^7^, ^8^, ^9^, ^10^ Indeed, the importance of shared decision‐making between patients and their providers in oncology care is becoming more recognized, and providers must navigate how they can best provide information to their patients needed to manage risks and benefits of various treatment and management options.^11^ When patients' level of involvement with their decision‐making matches their preferences, they experience less decision regret, further emphasizing the importance of involving patients in their care decisions.^12^

Molecular‐based technologies for predicting CM outcomes have been of recent interest. The validated 31‐gene expression profile test (31‐GEP) (Castle Biosciences, Inc.) that analyzes differential gene expression of a validated panel of 31 gene targets^13^, ^14^ has been available for clinical use since 2013 and clinical use has increased recently. 31‐GEP test results classify tumors as low (Class 1A), intermediate (Class 1B and 2A), or high risk (Class 2B) for metastasis to the sentinel lymph nodes, or for recurrence, distant metastasis or melanoma‐specific death 5 years after diagnosis of disease.^15^, ^16^, ^17^, ^18^, ^19^, ^20^, ^21^

The objective of the present study was to understand patients' attitudes toward prognostic testing generally and with 31‐GEP testing specifically, and to assess whether patients who had 31‐GEP testing experienced regret about their decision.

## 2 METHODS

### 2.1 Survey

A 43‐question online survey (Appendix S1) was distributed via email and newsletter by the Melanoma Research Foundation to individuals on their opt‐in communications list and made available from June 14, 2021 through August 2, 2021, to patients with Stage I–III CM. All patients enrolled in the Melanoma Research Foundation database were invited to participate in the online survey, regardless of whether they had been offered any prognostic testing at the time of diagnosis. Demographic questions were limited to avoid the potential for identification. The study captured anonymous information regarding patients' experiences and attitudes toward prognostic testing for CM generally and 31‐GEP testing specifically. Validated questions regarding shared decision‐making from the Shared Decision Making Process Scale were also included.^22^ The remaining questions that were not related to shared decision‐making or decision regret comprised questions included in previously published surveys and new questions developed and reviewed by a committee, including authors of the study.^23^, ^24^

The 31‐GEP test was made clinically available in 2013. To capture the first full year of clinical use, we only analyzed those responses in which participants self‐reported a melanoma diagnosis in or after 2014 (*n* = 120), at which time 31‐GEP prognostic testing became available, or if the respondent reported that they had received 31‐GEP testing and reported a test result.

Two hundred eighty‐one participants completed the survey. No identifying information about the participants was collected as part of the survey, and the survey was submitted to the Advarra IRB and deemed Exempt Human Subject Research by Advarra IRB.

### 2.2 Decision regret

Patients were asked questions regarding the decision to undergo 31‐GEP testing and the extent to which they experienced decision regret using a validated set of Decision Regret Scale questions.^25^, ^26^ Briefly, the decision regret scoring system uses a series of five questions scored on a scale of 1–5 that assess patients' attitudes about if: (1) the decision was the right one, (2) the decision was a good choice, (3) they would make the same choice again, (4) the decision harmed them, and (5) the decision was a wise choice. The scores for each of the five questions were averaged (mean) to calculate each respondent's total decision regret score. We considered a response of 1 or 2 to be little or no regret, 3 a neutral response, and 4 or 5 some or high regret.

### 2.3 Statistical analyses

The analysis was powered to detect a difference of 1.0 on the scale at 80% power and a family‐wise alpha of 0.05. Differences between responses were analyzed by Chi‐square test to determine statistical significance. Decision regret was assessed using the validated Decision Regret Scale, as previously described.^25^, ^26^ Differences in decision regret scores between patients with low‐ or high‐risk 31‐GEP results were compared using Wilcoxon rank sum test. Median decision regret scores for patients with low‐ or high‐risk 31‐GEP results were compared to a level of decision regret above neutral (>3) using Wilcoxon rank sum test. *p* < 0.05 were considered statistically significant.

## 3 RESULTS

### 3.1 Participant demographics

We included all responses for patients diagnosed in 2014 and later in our analyses (*n* = 120, 75.0% female). Participant demographics are shown in Table 1. Of the 120 participants analyzed, 75 indicated they did not receive 31‐GEP testing, 17 did not know if they had testing done, and 28 (78.6% female) indicated that they did receive 31‐GEP testing. Of the 28 participants who responded that they had 31‐GEP testing performed, four did not answer any survey questions following this question.

**TABLE 1:** Participant demographics

Demographics	Total in analysis, *n* (%) *n* = 120	Tested, *n* *n* = 28	Not tested, *n* *n* = 75	Unknown, *n* *n* = 17	Diagnosed before 2014^a^, *n* *n* = 161
Gender					
Male	29 (24.2)	6	23	–	46
Female	90 (75.0)	22	68	–	114
Prefer not to share	1 (0.8)	0	1	–	1
Year of diagnosis					
Before 2014	3 (2.5)	3			161
2014–2018	66 (55.0)	9	46	11	n/a
2019–2021	51 (42.5)	16	29	6	n/a
Did you have DecisionDx testing?					
Yes	28 (23.3)				1
No	75 (62.5)				138
Unsure	17 (14.2)				22
Insurance coverage					
Commercial	84 (70.0)	22	55	7	109
Commercial with MedAdvantage	6 (5.0)	2	4	0	1
Medicare	21 (17.5)	3	12	6	37
None	1 (0.8)	0	1	0	2
I do not know/did not answer	8(6.7)	0	1	1	12
Desired prognostic information about tumor					
Yes	108 (90.0)	27	67	14	134
No	7 (5.8)	0	6	1	10
Unsure	5 (4.2)	1	2	2	17

^a^ The 31‐GEP test became widely available in 2014. Therefore, we did not include patients who were diagnosed before this time and who did not state they received 31‐GEP (with additional responses) in the main analysis because the test would not likely have been available to these respondents.

### 3.2 Attitudes toward prognostic testing and 31‐GEP testing

Most respondents desired prognostic information about their tumors at diagnosis (*n* = 108, 90%) (Table 1). This was true whether or not the respondent had received 31‐GEP testing (*n* = 27 of those tested, 96.4% and *n* = 67 of those not‐tested, 89.3%, *p* = 0.288). Few patients reported that their provider had discussed 31‐GEP testing with them at the time of their diagnosis, including 11 patients who received 31‐GEP testing and five who did not receive 31‐GEP testing. Of respondents who did not have 31‐GEP testing, 53.8% wished they had been offered the option of 31‐GEP testing, 37.5% did not know, and 8.8% did not wish they had been offered testing (data not shown).

### 3.3 Attitudes of respondents receiving 31‐GEP testing

The distribution of 31‐GEP class results for patients who received 31‐GEP testing is shown in Table 2. The class distribution (Class 1A = 54.2%, Class 1B = 8.3%, Class 2A = 12.5%, and Class 2B = 12.5%) for respondents was similar to that reported in larger studies of patients.^27^

**TABLE 2:** GEP‐tested recipient questions

31‐GEP test recipients (*n* = 28)	Total, *n* (%)
31‐GEP class result	*n* = 24
Class 1A (low risk)	13 (54.2)
Class 1B (intermediate risk)	2 (8.3)
Class 2A (intermediate risk)	3 (12.5)
Class 2B (high risk)	3 (12.5)
Did not know/prefer not to share	3 (12.5)

How easy was it to interpret 31‐GEP results?	*n* = 24
Very easy	12 (50.0)
Somewhat easy	10 (41.7)
Difficult	1 (4.2)
Very difficult	1 (4.2)

How useful were the test results to you?	*n* = 24
Extremely useful	10 (41.7)
Useful	6 (25.0)
Somewhat useful	6 (25.0)
Not at all useful	2 (8.3)

**FIGURE 1:**
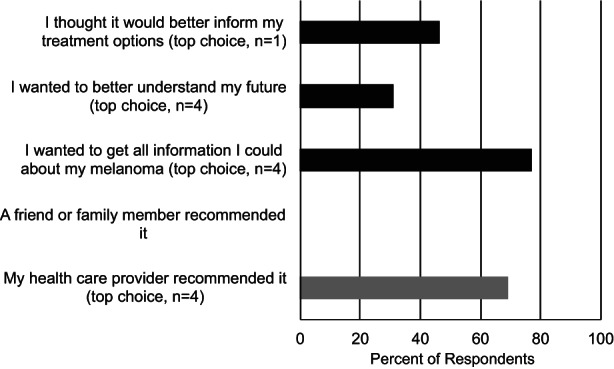
Reasons patients decided to have 31‐GEP testing. Respondents were asked to select the reasons that they chose to have 31‐GEP testing. Respondents were asked to choose all reasons that applied, and the graph indicates the percentage of respondents who selected each answer. Respondents were also asked to select their top reason for having testing, and the number of respondents who selected each reason as their top reason is shown in parentheses (top choice). Internally driven reasons are shown in black, and externally driven reasons are shown in gray (only respondents who answered that their healthcare provider asked if they wanted 31‐GEP testing were included, *n* = 13 respondents).

When asked what factors impacted their decision to get 31‐GEP testing, respondents stated they wanted all the information they could have about their tumor (76.9%), their healthcare provider recommended it (69.2%), they thought it would better inform their treatment decisions (46.2%), and they wanted to better understand their future (30.8%) (Figure 1). Of the 28 respondents who stated they had 31‐GEP testing, 13 stated that their provider had asked if they wanted 31‐GEP testing. Of these 13 respondents, when asked if they had any concerns about getting 31‐GEP testing, the most common response was that they did not have concerns (*n* = 5, 38.5%). Of those who had concerns, they included cost (*n* = 4, 30.8%), accuracy (*n* = 4, 30.8%), impact of poor prognosis on mental/emotional health (*n* = 3, 23.1%), and having sufficient tumor material available for other tests (*n* = 1, 7.7%) (data not shown).

To assess survivorship bias in the survey results, we compared the results of patients diagnosed at least 3 years previously (before 2019) with those diagnosed recently (between 2019 and 2021). These time frames were selected because adjuvant therapies and increased surveillance for CM often continue 2–3 years post‐diagnosis, and we wanted to reflect this in our comparison. Chi‐square analysis did not find significant differences between patient concerns for those diagnosed recently versus more than 3 years ago (*p* = 0.19).

**FIGURE 2:**
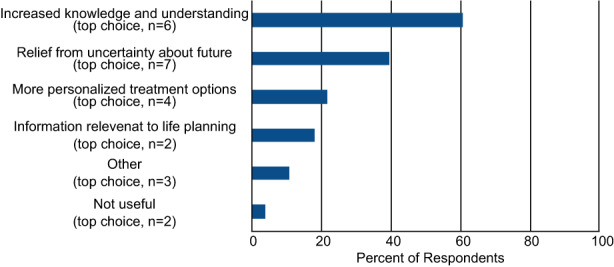
Benefits of 31‐GEP testing for patients. Respondents who received 31‐GEP testing were asked what benefits they felt they received from their 31‐GEP test results. Respondents were first asked to select all of the benefits they felt they gained with 31‐GEP testing (allowed to select as many responses as applied), and the percent of respondents that selected a given choice is shown. The patients were then asked to select what they most gained from the test results (select only one option), and the number of respondents who selected a particular option is indicated in parentheses (top choice).

Respondents generally felt that the test results were easy to understand; 22 out of 24 respondents stated the results were very or somewhat easy to understand, while 2 out of 24 stated the results were difficult or very difficult to understand (Table 2). Additionally, 22 out of 24 respondents felt the results were at least somewhat useful to them, and only 2 respondents felt the results were not useful at all (Table 2), and there were no differences in the responses based on the respondents' 31‐GEP class result. Respondents felt that their test results provided increased knowledge (60.7%), relief from uncertainty about the future (39.3%), more personalized treatment options (21.4%), information relevant to life planning (17.9%), or other unspecified value (10.7%) (Figure 2). Only 3.6% answered that the test results were not useful. Again, results were not significantly different between those diagnosed before 2019 and those diagnosed 2019–2021 (*p* = 0.30).

### 3.4 Decision regret

**FIGURE 3:**
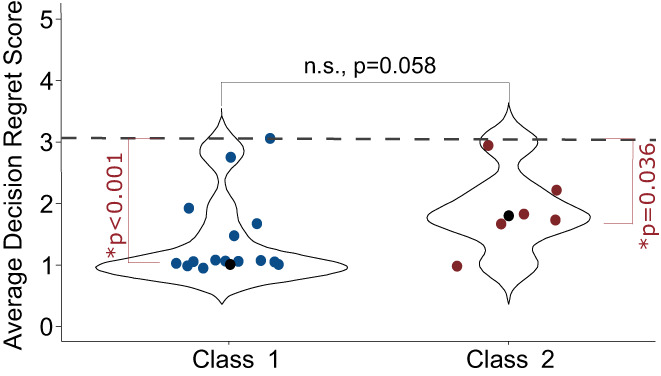
Decision Regret Scale Score. Respondents were asked a series of five validated questions that gage patients' level of regret regarding health care decisions. Questions are scored on a 1–5 scale, with answers of 1 or 2 indicating little or no regret, 3 being neutral, and 4 or 5 indicating some or high regret, and the answers were averaged. Averages <3.0 were considered to not have regret. Blue (Class 1) and red (Class 2) circles represent each respondent's mean decision regret score. Black circles indicate the median decision regret score for all Class 1 or Class 2 respondents. The dashed line indicates decision regret (3.05). *Statistically significant; n.s., not significant.

Twenty‐one respondents who received 31‐GEP testing answered the questions related to decision regret (*n* = 15, Class 1 and *n* = 6, Class 2). None of the respondents answered that they had increased (decision regret score of 4) or high (decision regret score of 5) levels of regret to any of the questions. Average individual decision regret scores were not significantly different between respondents who received Class 1 or Class 2 GEP test results (mean Class 1 = 1.39 and mean Class 2 = 1.90, *p* = 0.058) (Figure 3). Both groups' mean decision regret score was significantly less than a neutral score of 3.05 (*p* < 0.001, Class 1 and *p* = 0.036, Class 2), demonstrating that none of the respondents regretted their decision to undergo 31‐GEP testing. Because so few patients reported receiving a Class 2 result, we could not statistically analyze their responses for survivorship bias. However, we found no statistically significant differences in decision regret scores between patients diagnosed before 2019 and those diagnosed in 2019–2021 who received Class 1 results (*p* = 0.54) (data not shown).

## 4 DISCUSSION

The 31‐GEP test is a validated prognostic tool for treatment and surveillance planning for patients with CM.^27^, ^28^, ^29^, ^30^, ^31^ The majority of patients diagnosed with AJCC Stage I or II tumors have very good outcomes; however, some of these patients will experience recurrence and metastasis.^2^, ^3^ The 31‐GEP test accurately predicts the risk of recurrence independently from and in combination with AJCC staging, showing that it complements traditional clinicopathologic factors.^18^, ^19^, ^20^, ^32^ Previous studies have shown that the 31‐GEP results alter clinical management approximately 50% of the time.^28^, ^30^, ^31^

In our study, CM patients overwhelmingly desired prognostic information—both among those who received 31‐GEP testing and those who did not receive 31‐GEP testing. Patients felt that prognostic testing provided more information about their tumors and allowed them to make better treatment decisions and decisions about future planning. The most frequent response when asked if patients had any concerns about receiving 31‐GEP testing was that they did not have concerns, and those who did were concerned about the cost, accuracy, and the impact of a poor prognosis result on their emotional well‐being.

To better assess whether patients who received 31‐GEP testing regretted this choice, we asked a series of validated questions to obtain a decision regret score.^25^, ^26^ There was no significant difference between decision regret scores for patients with Class 1 versus Class 2 31‐GEP results. None of the respondents expressed regret, with the average decision regret score for respondents below the neutral regret score. Of note, even patients who received a high‐risk (Class 2) outcome from 31‐GEP testing did not regret their decision despite receiving a poor prognosis. This result is consistent with previous reports assessing decision regret in patients with other tumor types, including breast and prostate cancers, which found that patient‐related functional outcomes are not necessarily associated with increased decision regret.^6^, ^9^, ^33^ This is an important finding, given existing conjecture that patients who receive a high‐risk 31‐GEP test result may experience unnecessary anxiety or stress.^34^ This study shows that patient engagement through the addition of the 31‐GEP test has a positive impact on and is not regretted by patients.

### 4.1 Limitations

A limitation of the study is the small number of respondents who received 31‐GEP testing. Due to the anonymous nature of the survey and distribution list, we were unable to assess a response rate or confirm that respondents who self‐reported they had 31‐GEP testing did indeed receive the testing. Additionally, most of the respondents were female (75%), which is not surprising given that women are more likely to complete surveys than males.^35^ Of note, males make up 55% of those tested with the 31‐GEP.^1^ Finally, to assess potential survivorship bias, we compared the group of respondents diagnosed before 2019 with those diagnosed between 2019 and 2021 and found no statistically significant differences; however, the low number of respondents who had 31‐GEP testing made sub‐group statistical comparisons difficult.

### 4.2 Clinical implications

Our data suggest that patients are generally satisfied with their decision to get 31‐GEP testing, even when their result indicates a high risk of developing metastases. Indeed, respondents indicated that the results provided relief from uncertainty about the future and additional information relevant to life planning decisions, similar to patients who undergo genetic testing to assess the risk of familial melanoma or prognostic testing for uveal melanoma.^36^, ^37^ Others who have investigated decision regret in the context of cancer‐related choices found patient involvement with their medical decisions is associated with a higher level of satisfaction with the decision.^5^ Patients report more satisfaction with their decisions and higher quality of life when they felt that they had the information needed to make an informed decision and were active participants in the decision‐making process.^5^, ^7^, ^8^, ^9^, ^38^, ^39^, ^40^ The 31‐GEP test provides independent prognostic information that helps patients feel more informed and confident about their provider's suggested treatment and surveillance plans for their disease, thereby lessening regret.

## 5 CONCLUSIONS

Patients diagnosed with CM desire prognostic information about their tumors. Patients who received 31‐GEP testing felt that the 31‐GEP test relieved them of uncertainty, provided them important information to help with management‐ and treatment‐related decision‐making and planning for the future, and they did not regret their decision.

## AUTHOR CONTRIBUTIONS

Kelli Ahmed, Jennifer J. Siegel, and Kyleigh LiPira conceptualized the study; designed the survey, and collected survey data; Jennifer J. Siegel provided statistical support and data analysis; Sonia K. Morgan‐Linnell and Jennifer J. Siegel analyzed study data; Sonia K. Morgan‐Linnell wrote the original draft of the manuscript; and all authors critically reviewed and edited the manuscript. All authors have read and agreed to the published version of the manuscript.

## FUNDING INFORMATION

This study was funded by Castle Biosciences, Inc.

## CONFLICTS OF INTEREST

Kelli Ahmed, Jennifer J. Siegel, and Sonia K. Morgan‐Linnell are employees and options holders at Castle Biosciences, Inc. Kyleigh LiPira has no conflicts of interest to declare.

## ETHICS APPROVAL

The study received exemption from Institutional Review Board review by Advarra IRB (Columbia, MD).

## CONSENT

The study was considered exempt from written informed consent and IRB review by the Advarra IRB.

## Supporting information


Appendix S1

## Data Availability

The data are presented in the manuscript tables and figures. The authors certify that this manuscript reports original data. No additional data will be made available publicly.
