# Supplementary material for: Attitudes of patients with cutaneous melanoma toward prognostic testing using the 31‐gene expression profile test
Source: Cancer Med. 2022 Aug 1;12(2):2008–15. doi: 10.1002/cam4.5047 (PMC9883557; doi:10.1002/cam4.5047)
Supplement: Supplementary file 1 — Appendix S1 [file CAM4-12-2008-s001.docx]

Appendix A. Questions for 31-GEP Tested Respondents

**Questionnaire Part A:**

*When you were diagnosed with cutaneous melanoma, your doctor may have offered you prognostic testing to help predict the risk of your cancer spreading. This questionnaire is designed to help us better understand how patients feel about prognostic testing and their experiences as a cutaneous melanoma patient. The questionnaire should take 10-15 minutes to complete and should be completed honestly and without help from anyone else. Please answer all of the questions to the best of your ability.*

1. What was your sex at birth?
   1. Male
   2. Female
   3. Prefer not to share
2. What State did you live in when you were first diagnosed with cutaneous melanoma?
   1. _________________________________
3. What year were you first diagnosed with cutaneous melanoma?
   1. 2020
   2. 2019
   3. 2018
   4. 2017
   5. 2016
   6. 2015
   7. 2014
   8. Prior to 2014
4. At the time of your cutaneous melanoma diagnosis, did you want information about your prognosis (i.e., likelihood or risk for the cutaneous melanoma spreading or metastasizing)?
   1. Yes
   2. No
   3. I don’t know
5. Did you have the prognostic test called DecisionDx®-Melanoma done? *DecisionDx®-Melanoma is 31-gene expression profile test developed by Castle Biosciences, which reports associated metastatic risk as Class 1A, 1B, 2A or 2B.*
   1. Yes
   2. No
   3. I don’t know

(If answer a. is chosen, continue to question #8). If answer a. is not chosen, continue to **Questionnaire Part B**

*Please think about what happened when you talked with your healthcare providers including doctors, nurses, and other health care professionals about getting the DecisionDx-Melanoma test for your cutaneous melanoma prognosis.*

1. How much did you and your health care providers talk about the reasons you might want to have prognostic testing with DecisionDx-Melanoma?
   1. A lot
   2. Some
   3. A little
   4. Not at all
2. How much did you and your health care providers talk about the reasons you might not want to have prognostic testing with DecisionDx-Melanoma?
   1. A lot
   2. Some
   3. A little
   4. Not at all
3. Did any of your health care providers talk about alternative prognostic tests (other than DecisionDx-Melanoma) as something that you should seriously consider?
   1. Yes
   2. No
4. Did any of your health care providers ask if you wanted to have prognostic testing with DecisionDx-Melanoma?
   1. Yes
   2. No
5. How did you *first* find out about the DecisionDx-Melanoma test offered by Castle Biosciences?
   1. My doctor (or other healthcare provider) told me about it
   2. A friend or family member recommended it
   3. I found it through an online search
   4. Other: ___________________________________________________________________
6. Why did you decide to get the DecisionDx-Melanoma test? (choose *all* that apply)
   1. My doctor (or other healthcare provider) recommended it
   2. A friend or family member recommended it
   3. I wanted to get all information I could about my melanoma
   4. I thought it would better inform my treatment options
   5. I wanted to better understand my future
   6. Other: ___________________________________________________________________
7. Which of the following **MOST** impacted your decision to get the test (select only *one*)
   1. My doctor (or other healthcare provider) recommended it
   2. A friend or family member recommended it
   3. I wanted to get all information I could about my melanoma
   4. I thought it would better inform my treatment options
   5. I wanted to better understand my future
   6. Other: ________________________________________________________________________
8. Did you (or a family member) research DecisionDx-Melanoma online prior to making your decision to get the test?
   1. Yes
   2. No
   3. I don’t know
9. What were your concerns (if any) about getting prognostic testing with DecisionDx-Melanoma? (select *all* that apply)
   1. I was concerned about the impact that a poor prognosis would have on my mental/emotional health
   2. I was concerned about the accuracy of the test
   3. I was concerned about the cost of the test
   4. Other:__________________________________________________________________
   5. I didn’t have any concerns
10. What concerned you the **MOST** (if anything) about getting prognostic testing with DecisionDx-Melanoma? (select only *one*)
    1. I was concerned about the impact that a poor prognosis would have on my mental/emotional health
    2. I was concerned about the accuracy of the test
    3. I was concerned about the cost of the test
    4. Other:__________________________________________________________________
    5. I didn’t have any concerns
11. What was your DecisionDx-Melanoma test result?
    1. Class 1A
    2. Class 1B
    3. Class 2A
    4. Class 2B
    5. My sample failed to generate a test result
    6. I don’t know, or prefer not to share
12. Was your doctor able to answer all your questions about the results of your test?

My doctor:

- 1. answered all of my questions
  2. answered most of my questions
  3. answered some of my questions
  4. was not able to answer any of my questions
  5. I didn’t have any questions

1. How easy was it for you to understand your result (Class 1A, Class 1B, Class 2A, or Class 2B) from the test report?
   1. Very easy
   2. Somewhat easy
   3. Difficult
   4. Very difficult
2. How useful were the test results to you?
   1. Extremely useful
   2. Useful
   3. Somewhat useful
   4. Not at all useful

Describe HOW this information has been useful to you. ____________________________________________________________________________________________________________________________________________________________________________________________________________________________________________________________________________________

1. What do you feel you have gained from your test results? (Select *all* that apply)
   1. Increased knowledge and understanding
   2. Relief from uncertainty about the future
   3. More personalized treatment options
   4. Information relevant to life planning
   5. Other reasons not listed above.

(Please describe): ______________________________________________________

- 1. NA-test results are not useful

1. What do you feel you have gained **MOST** from your test results? (Select *one*)
   1. Increased knowledge and understanding
   2. Relief from uncertainty about future
   3. More personalized treatment options
   4. Information relevant to life planning
   5. Other:

Please describe: ________________________________________________________

- 1. NA-test results are not useful

*Please think about the decision you made to get the DecisionDx-Melanoma* *test after talking to your doctor.*

1. It was the right decision.
   1. Strongly agree
   2. Agree
   3. Neither agree nor disagree
   4. Disagree
   5. Strongly disagree
2. I regret the choice that was made.
   1. Strongly agree
   2. Agree
   3. Neither agree nor disagree
   4. Disagree
   5. Strongly disagree
3. I would make the same choice if I had to do it over again.
   1. Strongly agree
   2. Agree
   3. Neither agree nor disagree
   4. Disagree
   5. Strongly disagree
4. The choice did me a lot of harm.
   1. Strongly agree
   2. Agree
   3. Neither agree nor disagree
   4. Disagree
   5. Strongly disagree
5. The decision was a wise one.
   1. Strongly agree
   2. Agree
   3. Neither agree nor disagree
   4. Disagree
   5. Strongly disagree
6. Do you feel like COVID-19 impacted your follow-up care for melanoma at all?
   1. Yes, I have not been to the doctor as often
   2. Yes, my doctor has switched to virtual or telemedicine visits instead of in-person
   3. Yes-Other
      1. Please describe
   4. No, I have kept all of my appointments
   5. No, I no longer receive follow-up care for melanoma
   6. No, other
      1. Please describe

(Optional) Please provide a detailed explanation of how COVID-19 has changed or not changed your melanoma treatment.

(Optional) Please provide a detailed explanation of how COVID-19 has changed or not changed your melanoma treatment.

1. Do you feel like COVID-19 impacted your follow-up care for melanoma at all?
   1. Yes, I have not been to the doctor as often
   2. Yes, my doctor has switched to virtual or telemedicine visits instead of in-person
   3. Yes-Other
      1. Please describe
   4. No, I have kept all of my appointments
   5. No, I no longer receive follow-up care for melanoma
   6. No, other
      1. Please describe

(Optional) Please provide a detailed explanation of how COVID-19 has changed or not changed your melanoma treatment.

1. (Optional) Was your care (biopsy/ wide local excision/sentinel lymph node biopsy) affected/delayed by COVID?
   1. Yes
   2. No
   3. I don’t know
2. (Optional) What type of insurance coverage do you have?
   1. Medicare
   2. Commercial with MedAdvantage
   3. Commercial
   4. I don’t have insurance
   5. I don’t know

Appendix B. Questions for non-31-GEP Tested Respondents

**Questionnaire Part A:**

*When you were diagnosed with cutaneous melanoma, your doctor may have offered you prognostic testing to help predict the risk of your cancer spreading. This questionnaire is designed to help us better understand how patients feel about prognostic testing and their experiences as a cutaneous melanoma patient. The questionnaire should take 10-15 minutes to complete and should be completed honestly and without help from anyone else. Please answer all of the questions to the best of your ability.*

1. What was your sex at birth?
   1. Male
   2. Female
   3. Prefer not to share
2. What State did you live in when you were first diagnosed with cutaneous melanoma?
   1. _________________________________
3. What year were you first diagnosed with cutaneous melanoma?
   1. 2020
   2. 2019
   3. 2018
   4. 2017
   5. 2016
   6. 2015
   7. 2014
   8. Prior to 2014
4. At the time of your cutaneous melanoma diagnosis, did you want information about your prognosis (i.e., likelihood or risk for the cutaneous melanoma spreading or metastasizing)?
   1. Yes
   2. No
   3. I don’t know
5. Did you have the prognostic test called DecisionDx®-Melanoma done? *DecisionDx®-Melanoma is 31-gene expression profile test developed by Castle Biosciences, which reports associated metastatic risk as Class 1A, 1B, 2A or 2B.*
   1. Yes
   2. No
   3. I don’t know

If answer a. is not chosen, continue to **Questionnaire Part B**

**Questionnaire Part B: for patients that did not receive testing with DecisionDx-Melanoma.**

*DecisionDx-Melanoma (Castle Biosciences) is a molecular test that predicts the risk that your tumor will metastasize (spread). The accuracy of this test is supported by over 10 years of scientific research. Please think about what happened when you talked with your healthcare providers (including doctors, nurses, and other health care professionals) about your treatment plan, including prognostic testing with the DecisionDx-Melanoma test.*

1. How much did you and your doctor talk about the reasons you might want to have prognostic testing done with DecisionDx-Melanoma?
   1. A lot
   2. Some
   3. A little
   4. Not at all
2. How much did you and your doctor talk about the reasons you might not want to have prognostic testing done with DecisionDx-Melanoma?
   1. A lot
   2. Some
   3. A little
   4. Not at all
3. Did your doctor talk about any alternative prognostic testing (other than DecisionDx-Melanoma) as something that you should seriously consider?
   1. Yes
   2. No
4. Did your doctor ask if you wanted to have prognostic testing done with DecisionDx-Melanoma to help determine your risk of metastasis?
   1. Yes
   2. No

If YES to #9:

1. Why did you decide not to have the DecisionDx-Melanoma test done (select *all* that apply)?
   1. I was concerned about getting a high-risk result
   2. I was concerned about the accuracy of the test
   3. I was concerned about the cost of the test
   4. I decided I did not want to know my prognosis
   5. Other:______________________________________________________________
2. What **MOST** influenced your decision not to have the DecisionDx-Melanoma test done (select only *one*)
   1. I was concerned about getting a high-risk result
   2. I was concerned about the accuracy of the test
   3. I was concerned about the cost of the test
   4. I decided I did not want to know my prognosis
   5. I chose an alternative prognostic test
   6. Other:______________________________________________________________

*Please think about the decision you made to not get the DecisionDx-Melanoma test after talking to your doctor.*

1. It was the right decision.
   1. Strongly agree
   2. Agree
   3. Neither agree nor disagree
   4. Disagree
   5. Strongly disagree
2. I regret the choice that was made.
   1. Strongly agree
   2. Agree
   3. Neither agree nor disagree
   4. Disagree
   5. Strongly disagree
3. I would make the same choice if I had to do it over again.
   1. Strongly agree
   2. Agree
   3. Neither agree nor disagree
   4. Disagree
   5. Strongly disagree
4. The choice did me a lot of harm.
   1. Strongly agree
   2. Agree
   3. Neither agree nor disagree
   4. Disagree
   5. Strongly disagree
5. The decision was a wise one.
   1. Strongly agree
   2. Agree
   3. Neither agree nor disagree
   4. Disagree
   5. Strongly disagree
6. Do you feel like COVID-19 impacted your follow-up care for melanoma at all?
   1. Yes, I have not been to the doctor as often
   2. Yes, my doctor has switched to virtual or telemedicine visits instead of in-person
   3. Yes-Other
      1. Please describe
   4. No, I have kept all of my appointments
   5. No, I no longer receive follow-up care for melanoma
   6. No, other
      1. Please describe

(Optional) Please provide a detailed explanation of how COVID-19 has changed or not changed your melanoma treatment.

If NO to #9:

1. Do you wish your doctor had offered you the option of testing with DecisionDx-Melanoma to better understand your prognosis?
   1. Yes
   2. No
   3. I don’t know
2. Do you feel like COVID-19 impacted your follow-up care for melanoma at all?
   1. Yes, I have not been to the doctor as often
   2. Yes, my doctor has switched to virtual or telemedicine visits instead of in-person
   3. Yes-Other
      1. Please describe
   4. No, I have kept all of my appointments
   5. No, I no longer receive follow-up care for melanoma
   6. No, other
      1. Please describe

(Optional) Please provide a detailed explanation of how COVID-19 has changed or not changed your melanoma treatment.

1. (Optional) Was your care (biopsy/ wide local excision/sentinel lymph node biopsy) affected/delayed by COVID?
   1. Yes
   2. No
   3. I don’t know
2. (Optional) What type of insurance coverage do you have?
   1. Medicare
   2. Commercial with MedAdvantage
   3. Commercial
   4. I don’t have insurance
   5. I don’t know
